# Effects of Ecologically Relevant Concentrations of Cadmium on the Microbiota, Short-Chain Fatty Acids, and FFAR_2_ Expression in Zebrafish

**DOI:** 10.3390/metabo13050657

**Published:** 2023-05-15

**Authors:** Jian Yang, Junyi Li, Xiaoshun Zhang, Qin Zhou, Junyi Wang, Qingsong Chen, Xiaojing Meng, Yuan Xia

**Affiliations:** 1 School of Public Health, Guangdong Pharmaceutical University, 283, Jianghaidadao, Guangzhou 510006, China; 2 Guangdong Provincial Key Laboratory of Tropical Disease Research, Department of Occupational Health and Occupational Medicine, School of Public Health, Southern Medical University, Guangzhou 510515, China

**Keywords:** cadmium, zebrafish, microbiota, SCFAs, FFAR_2_

## Abstract

Exposure to cadmium (Cd) can affect neurodevelopment and results in increased potential of developing neurodegenerative diseases during the early developmental stage of organisms, but the mechanisms through which exposure to environmentally relevant concentrations of Cd lead to developmental neurotoxicity remain unclear. Although we know that microbial community fixations overlap with the neurodevelopmental window during early development and that Cd-induced neurodevelopmental toxicity may be related to the disruption of microorganisms during early development, information on the effects of exposure to environmentally relevant Cd concentrations on gut microbiota disruption and neurodevelopment is scarce. Therefore, we established a model of zebrafish exposed to Cd (5 µg/L) to observe the changes in the gut microbiota, SCFAs, and free fatty acid receptor 2 (FFAR_2_) in zebrafish larvae exposed to Cd for 7 days. Our results indicated that there were significant changes in the gut microbial composition due to the exposure to Cd in zebrafish larvae. At the genus level, there were decreases in the relative abundances of *Phascolarctobacterium*, *Candidatus Saccharimonas*, and *Blautia* in the Cd group. Our analysis revealed that the acetic acid concentration was decreased (*p* > 0.05) while the isobutyric acid concentration was increased (*p* < 0.05). Further correlation analysis indicated a positive correlation between the content of acetic acid and the relative abundances of *Phascolarctobacterium* and *Candidatus Saccharimonas* (*R* = 0.842, *p* < 0.01; *R* = 0.767, *p* < 0.01), and a negative correlation between that of isobutyric acid and the relative abundance of *Blautia glucerasea* (*R* = −0.673, *p* < 0.05). FFAR_2_ needs to be activated by SCFAs to exert physiological effects, and acetic acid is its main ligand. The FFAR_2_ expression and the acetic acid concentration were decreased in the Cd group. We speculate that FFAR_2_ may be implicated in the regulatory mechanism of the gut–brain axis in Cd-induced neurodevelopmental toxicity.

## 1. Introduction

Cadmium (Cd), a highly toxic heavy metal pollutant, is widely available in the environment. Because of its long half-life (about 20–40 years) and slow excretion rate, Cd tends to accumulate in the human body, leading to various toxic effects, the most concerning of which is developmental neurotoxicity, which includes the induction of neuronal apoptosis, glial cell activation and neuroinflammatory factor release in the brain nervous system. China is among the countries with serious Cd pollution, and there have been several cases of Cd pollution in Chinese river basins. For example, the concentration of Cd in freshwater is usually between 10 and 500 ng/L, while the Cd concentration in the Luan River basin in northeastern China is 1.120–4.474 µg/L (the national drinking water standard (GB/T5750-2006) is 5 µg/L) and in the Longjiang River is 45.01 µg/L [1]. Most existing related studies explored Cd toxicity caused by high concentrations (100 mg/kg) in humans [2]. However, these concentrations are much higher than the Cd concentrations that the normal population is typically exposed to. According to Feng et al., low doses of Cd exposure can also cause various adverse effects [3,4]. However, the concentrations of Cd exposure used in existing studies (2.5 mg/L a week) are still much higher than the actual environmental exposure doses [5,6], and research related to environmental low-dose Cd exposure remains scarce. Our previous studies found that environmental low-concentration Cd exposure caused developmental neurotoxicity and changes in the gut microbiota of zebrafish [7], but the mechanisms involved were not explored in depth. It is hypothesized that the gut–brain axis may be involved in the regulation of Cd-induced neurodevelopmental toxicity.

The microbiota has a crucial influence in maintaining normal physiological functions; notably, the gut–brain axis is a channel of interconnection between the gut and the brain [8]. The homeostasis of the brain environment and its downstream regulatory effects are more sensitive to the gut microbiota, and when the composition of gut microbiota changes significantly, metabolic processes in the brain are altered, leading to cognitive and behavioral dysfunction. The changing composition of the gut microbiota during homeostasis allows the release of metabolites synthesized by the microbiota into the gut lumen with effects on metabolism, immune system function and behavior. Heavy metal exposure leads to changes in the composition of the gut flora and metabolites, which in turn affect the integrity of the gut barrier, and the destruction of the gut barrier leads to an increased intake of heavy metals in the body, thus further enhancing their toxicity; at the same time, changes in intestinal metabolites can affect the function of the central nervous system through the gut–brain axis. Exposure to chemicals during the window of embryonic and early larval development, even at low concentrations considered safe, can disrupt the intestinal flora [9]. The animal colon contains large numbers of undigested complex carbohydrates (oligosaccharides, non-starch polysaccharides, resistant starch, etc.), which are able to serve as substrates for anaerobic fermentation by intestinal bacteria, and the main metabolic end products produced are short-chain fatty acids (SCFAs), which are the most important metabolites of the intestinal microbiota, especially acetate, propionate and butyrate, while increased levels of intestinal SCFAs can regulate intestinal flora balance and mitigate cognitive dysfunction and are now recognized as the link between the gut flora and the host cells; these molecules are made up of components of gut bacteria. Not only are they important energy sources that can be directly used by intestinal bacteria, but they are also one of the energy sources required by the intestinal epithelial cells. Of course, in addition to being a substrate for energy synthesis, the regulatory functions of SCFAs in host physiological activities and the immune response are beginning to be known. It is noteworthy that the physiological regulation of neurodevelopmental toxicity that SCFAs participate in requires the activation of the FFAR_2_-signaling pathway [10]. FFAR_2_ can be activated by a variety of SCFAs, but it is the most sensitive to acetic acid. Acetic acid is the major ligand of FFAR_2_ [11]; FFAR_2_ binding to acetic acid plays a crucial role in humans.

We examined the effects of Cd at 0.1, 1.25, 2.5, and 5 μg/L on zebrafish in our previous study [7] and found that the effects of Cd at 5 μg/L on neurodevelopmental toxicity and gut microbiota of zebrafish were significant. Therefore, in order to further explore the regulatory processes of the gut–brain axis in Cd neurodevelopmental toxicity, an experiment was conducted to examine the changes in the gut microbiota structure, SCFAs, and FFAR_2_ expression through exposing zebrafish embryos and larvae to Cd concentrations (5 µg/L) for 7 days and performing correlation analysis. Our results contribute to the neurodevelopmental toxicity mechanism of Cd and provide a new direction and reference basis for evaluating the environmental ecotoxicity of Cd.

## 2. Materials and Methods

### 2.1. Animals and Embryo Breeding

Wild-type AB-strain zebrafish were used in this experiment, which were purchased from the laboratory of the College of Public Health, Southern Medical University. Adult zebrafish were housed in tanks in a 14 h light and 10 h dark cycle at 28 ± 1 °C and a pH of 6.5–7.5. The zebrafish were fed twice a day, and the impurities in the fish tank were regularly cleaned during the experiment. Sexually mature male and female zebrafish were selected in a 1:1 ratio and placed in a tank with a partition for ovulation and fertilization. The next morning, the spacer was withdrawn, the embryos were collected within 30 min, using the zebrafish embryo growth medium used by Bahuguna et al. [12], and normal embryo was selected and maintained in Danieau’s embryo medium (17.4 mmol/L NaCl, 0.21 mmol/L KCl, 0.12 mmol/L MgSO4, 0.15 mmol/L, Ca(NO3)2, and 1.5 mmol/L HEPES; pH = 7.2), incubated in a constant temperature incubator at 28.5 °C. The fish were observed under a somatic microscope 2 h post-fertilization (hpf). After each group of zebrafish larvae had developed up until 5 days post-fertilization (dpf), they were placed in transparent water tanks and continued to be cultured, with 7 dpf as the observation endpoint to observe and record the developmental changes in zebrafish larvae.

### 2.2. Embryo Treatment

The concentration of heavy metals was calculated by the following equation. An amount of 0.07332 g of cadmium chloride was weighed and placed in a 50 mL centrifuge tube, while 40 mL of double distilled water (ddH_2_O) was measured with a measuring cylinder, added into the centrifuge tube, shaken well and allowed to stand to obtain 10 mmol/L of the CdCl_2_ transparent stock solution. The desired concentration (5 μg/L for Cd) was then obtained by adding 5 mL of the stock solution to 107 mL of ddH_2_O. The experiment included a control group and a Cd group, with 5 samples in each group and 40 zebrafish embryos in each sample. The collected embryos were maintained in Danieau’s embryo medium. Zebrafish embryos were exposed with Cd by adding 30 mL of 5 μg/L CdCl_2_ to Danieau’s embryo medium; after 12 h, impurities and dead embryos were removed. The control group was maintained without any Cd treatment.

$$ppm\;of\;element=\frac{Molecular\;weight\;of\;salts}{Molecular\;mass\;of\;the\;element}\times required\;Conc.\;of\;elements$$

### 2.3. Microbiota Analysis

Each group had 5 replicates with 40 larvae per replicate. Genomic DNA was extracted according to the instructions of DNA extraction kits corresponding to various samples. Then, the integrity and purity were tested by 1% agarose gel electrophoresis and NanoDropOne. Genomic DNA was used as a template for PCR amplification and the electrophoresis detection of products. Barcode primers and PremixTaq (TaKaRa) were used for PCR amplification according to the V3/V4 region. Illumina MiSeq sequencing was conducted at Magigene Co., Ltd. (Guangzhou, China). The QIIME data analysis package was used to perform 16S rRNA data analysis. The correlation matrix between bacterial microbiota composition and locomotor activity (activity counts and distance) was generated using Pearson’s correlation coefficient. A heat map of this correlation matrix was created using the pheatmap package in R software.

### 2.4. SCFAs Quantification by GC-MS

The larvae (7 dpf, n = 200 per group, five replicates) were transferred into the tube and washed at least three times with purified water. Then, the larvae were pooled and esterification using PBS and a ribitol internal standard was carried out. Afterward, the mixture was centrifuged at 4000 r/min for 10 min. The supernatant was aspirated and analyzed via GC-MS, and the concentration of SCFAs in zebrafish larvae was calculated.

### 2.5. FFAR_2_ Expression Examination via qPCR

The reagent trizol was added to the samples to extract the total RNA, followed by the addition of chloroform and then centrifugation. The isolated RNA was then precipitated using isopropyl alcohol, rinsed with ethanol, and detected using qPCR. The PCR conditions were as follows: 30 s incubation at 95 °C, followed by 40 cycles of 95 °C for 15 s, 55 °C for 20 s, and 72 °C for 20 s. Primers for FFAR_2_ and glyceraldehyde 3-phosphate dehydrogenase (gapdh) were generated using Primer3 software, as listed in Table 1.

### 2.6. Preparation of Graphics and Statistical Analysis

The results of quantitative resources were expressed as the mean ± standard deviation; graphics and statistical analysis were processed using SPSS16.0 software. Significant difference were distinguished at probability (*p*) value of <0.05.

## 3. Results

### 3.1. Alterations in the Structure of the Gut Microbiota in Zebrafish Larvae

**Figure 1:**
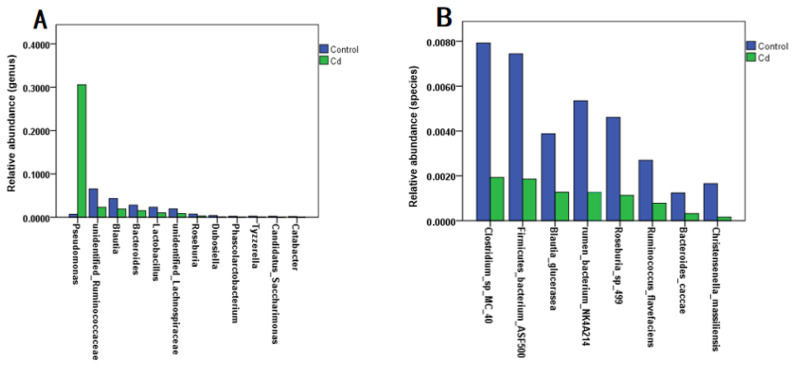
Effect of Cd exposure on the relative abundance of zebrafish gut microbiota. (**A**) Genus level; (**B**) species level.

Evidence shows that heavy metal exposure can disrupt the intestinal flora, affecting heavy metal absorption and ultimately enhancing or mitigating heavy metal toxicity [13]. To explored the effect of Cd exposure on the relative abundance of gut microbiota, we analyzed zebrafish larvae and found that Cd exposure caused a significant increase in the relative abundance of *Pseudomonas* at the genus level compared with the control group (*p* < 0.05). At the genus level, the relative abundances of gut microbiota in unidentified *Ruminococcaceae*, *Blauti*, *Bacteroides*, *Lactobacillus*, unidentified *Lachnospiraceae*, *Roseburia*, and *Dubosiella*, and *Phascolarctobacterium* were significantly decreased (*p* < 0.05) (Figure 1A). At the species level, Cd exposure caused a significant decrease in the relative abundances of *Clostridium* sp. MC 40, *Firmicutes* bacterium ASF500, *Blautia glucerasea*, rumen bacterium NK4A214, *Roseburia* sp. 499, *Ruminococcus flavefaciens*, *Bacteroides caccae*, and *Christensenella massiliensis* (*p* < 0.05) (Figure 1B). This was consistent with the results of the previous experiment [7].

### 3.2. Changes in the Concentrations of SCFAs in Zebrafish Larvae

**Figure 2:**
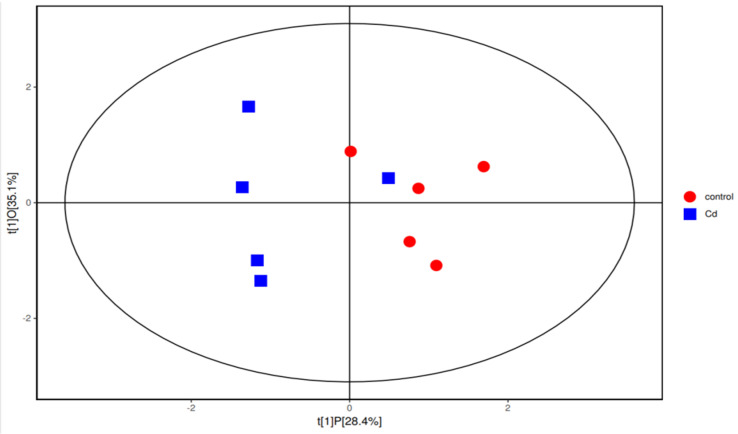
Orthogonal partial least-squares discrimination analysis (OPLS−DA) in the Cd group and the control group.

Gut flora-producing SCFAs include *Bacteroides*, *Lactobacillus*, and unidentified *Lachnospiraceae* [14]. Interestingly, the abundances of all these bacteria decreased in the Cd group in this study. To explore the mechanism of Cd-induced changes in the concentrations of SCFAs, the SCFAs were analyzed. The analysis showed that the average concentration of isobutyric acid in the Cd group was significantly increased (*p* < 0.05) (Table 2). The average concentrations of acetic acid between the control group and the Cd group showed no significant difference *(p* > 0.05). In addition, the mean concentrations of isovalerate, valerate, and caproic acid in SCFAs between the two groups showed no significant differences (*p* > 0.05). To further explore the relationship between Cd and SCFAs, we used orthogonal partial least-squares discrimination analysis (OPLS-DA) and found that the concentrations of SCFAs in the Cd group and the control group were different (Figure 2).

### 3.3. Correlation Analysis between SCFAs and Gut Flora

**Figure 3:**
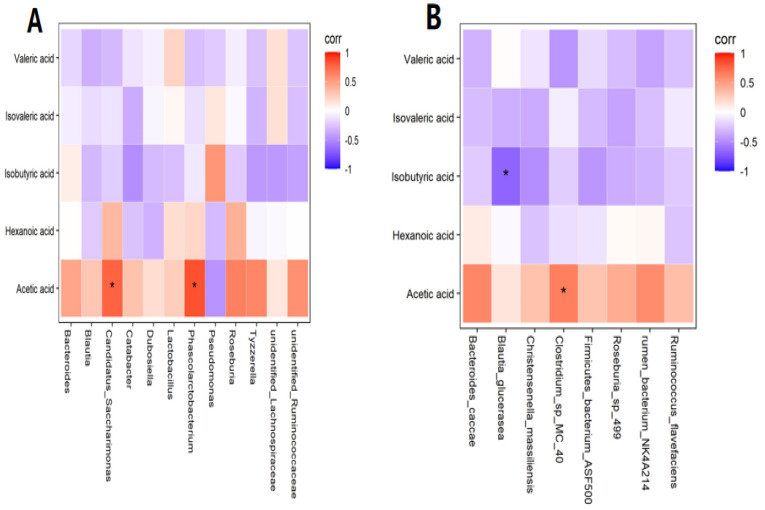
(**A**) Correlation analysis with short−chain fatty acids (SCFAs) at the genus level. (**B**) Correlation analysis with SCFAs at the species level. Note: * means significance at *p* < 0.05.

In preliminary experiments, we found some interesting phenomena. Although there was no statistical significance in the average concentrations of acetic acid in zebrafish larvae in the control group and Cd group (*p* > 0.05), the average concentration of acetic acid in the Cd group was 39.953 nmol/g, while that in the control group was 51.844 nmol/g. The concentration difference reached 11.981 nmol/g, which was a great difference. Therefore, further correlation analysis was conducted. At the genus level, a positive correlation was found between acetic acid and *Phascolarctobacterium* and *Candidatus Saccharimonas* (*R* = 0.842, *p* < 0.01; *R* = 0.767, *p* < 0.01) (Figure 3A). At the species level, a positive correlation was observed between acetic acid and *Clostridium* sp. *MC 40* in the Cd group (*R* = 0.648, *p* < 0.05), while a negative correlation was observed between isobutyric acid and *Blautia glucerasea* (*R* = −0.673, *p* < 0.05) (Figure 3B). Collectively, the changes in the concentrations of SCFAs were caused by changes in the gut flora.

### 3.4. Effects of Cd Exposure on Host Gene Expression

**Figure 4:**
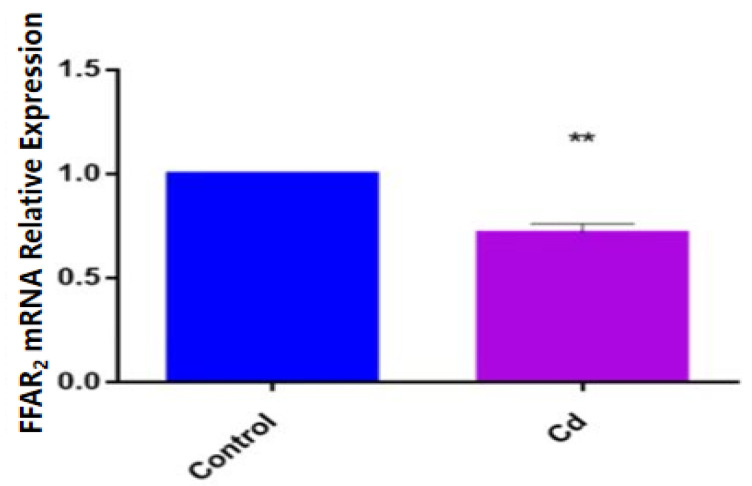
Relative expression of FFAR_2_ mRNA in zebrafish in the Cd group and control group. Note: ** means significance at *p* < 0.01.

To determine how the expression of FFAR_2_ changed under Cd exposure, we detected the expression of FFAR_2_ in zebrafish larvae via qPCR and found that the expression of FFAR_2_ mRNA in the Cd group was significantly decreased compared with that in the control group (*p* < 0.01) (Figure 4).

## 4. Discussion

Cd is one of the most toxic heavy metals, as well as a global pollutant and widely found in plastic manufacturing, metal smelting, battery, paint and other industries. Cd can damage human neurons and affect the construction of the neural network system and the development of synapses, thus causing cognitive impairment of neurological functions; the impact on children is especially of great concern. Long-term exposure to Cd can cause memory loss, inattention and even affect psychological behavior in children. Low-dose Cd exposure can cause neural-tube malformations and developmental delays in the early developmental stage. There is increasing evidence emphasizing the role of the gut’s flora and its metabolites in neurodevelopmental toxicity. The effects on the central nervous system could pass through the gut–brain axis [15,16]. Dysbiosis of the gut microflora can cause changes in the concentrations of metabolites, which can induce various diseases [17,18,19,20,21,22]. In this study, we established a zebrafish model, exposed it to environmentally relevant concentrations of Cd (5 µg/L), and examined the relevant indicators. In addition, we analyzed whether or not environmentally relevant Cd exposure altered the gut microbiota’s composition and the concentrations of SCFAs and subsequently investigated the effects of changes in the concentration of SCFAs on the expression of FFAR_2_ at the genetic level. Our results indicated that environmentally relevant concentrations of Cd had adverse effects on the gut flora and neuromotor behavior of zebrafish, resulting in the disruption of the intestinal flora, decreased concentration of SCFAs and reduced motility. In addition, our results indicated the changes in FFAR_2_ expression. It is worth focusing on the fact that the gut microbiota’s composition in zebrafish larvae was disturbed even when exposed to low concentrations of Cd (Figure 1A,B). The dominant groups in the zebrafish gut flora included *Firmicutes* and *Proteobacteria*, and the results indicated that the proportion of *Firmicutes* decreased while the proportion of *Proteobacteria* increased in zebrafish larvae after 7 days of Cd exposure. The above results were consistent with those of some existing studies [7,22].

To investigate the effect of gut microbial disruption on metabolites, this work examined the SCFAs in zebrafish larvae using gas chromatography–mass spectrometry. The results indicated that the disruption of the gut microbiota led to an increase in the concentrations of isobutyric acid (*p* < 0.05) and decrease in the concentrations of acetic acid (*p* > 0.05) in zebrafish. The concentrations of isobutyric acid are associated with some bacteria involved in mood regulation [23]. In the present study, further analysis revealed that the concentrations of isobutyric acid were negatively correlated with *Blautia glucerasea* (*R* = −0.673, *p* < 0.05). Petrov et al. indicated that the relative abundance of *Blautia glucerasea* in both patients with major depression disorders and Parkinson’s disease was decreased [24,25], suggesting that *Blautia glucerasea* is associated with psychiatric and neurodegenerative diseases. A related study from Belgium in 2016 [23] reported elevated isobutyric acid in the stools of 113 children with affective disorders, suggesting that changes in isobutyric acid concentrations may be related to children’s emotions. Three clinical studies showed significant differences in the composition of oropharyngeal [26,27] and intestinal flora [28] between patients with schizophrenia and control patients, and similar changes in intestinal bacterial profiles in children with autism were observed. Notably, the experimental results in this study showed a great difference in the mean concentration of acetic acid in the control group and the Cd group, with a decrease in the concentration of acetic acid in zebrafish from the Cd group compared with that in the control group. Acetic acid, as one of the most abundant SCFAs in the colon, is involved in many physiological modulations through regulating the levels of hypothalamic neurotransmitters, glutamate, and glutamine [29]. The difference in acetic acid concentrations was not statistically significant (*p* > 0.05) between the control group and Cd group, but the results are still important. In a study of depressed patients that found acetic acid concentrations to be significantly lower in depressed patients, the levels of SCFAs were also reduced in mice with Alzheimer’s disease (AD). The oral administration of acetate reduced cognitive dysfunction and improved neurological impairment in mice [29]. To further investigate the mechanisms involved, this study analyzed the correlation between intestinal flora and acetic acid. The test results indicated that the concentrations of acetic acid in the Cd group were highly significantly and positively correlated with the levels of both *Phascolarctobacterium* and *Candidatus Saccharimonas*. (*R* = 0.842, *p* < 0.01; *R* = 0.767, *p* < 0.01). *Phascolarctobacterium* belongs to *Firmicutes* and produces large amounts of acetic acid/propionic acid [30]. It is positively correlated with positive emotions in humans [31]. A large amount of evidence showed that *Phascolarctobacterium* has beneficial neuroprotective effects on the host [32], assists in improving the cognitive level, and reduces the prevalence of Alzheimer’s disease [33,34,35]. *Saccharimonas* was found to be abundant in the gut of patients with nonviolent schizophrenia, which provides new insights into violent behavior in schizophrenia from the perspective of gut microbes [36]. There is growing evidence that changes in gut microbial composition are associated with various neuropsychiatric disorders. From Figure 1, we can observe that, at the genus level, the relative abundances of eight gut microbes that may be connected with neurodevelopment were significantly lower in the Cd group than in the control group. This result suggests that Cd may affect neurodevelopment through the gut–brain axis channel. 

FFAR_2_ acts mainly by sensing SCFAs in the intestine and being activated by three major SCFAs: acetic acid, propionic acid, and butyric acid, with acetic acid being the primary ligand for FFAR_2_. The activation of FFAR_2_ by SCFAs is necessary for a normal resolution of certain inflammatory responses. Binding of FFAR_2_ by SCFAs may provide a molecular link between diet, gastrointestinal bacterial metabolism, immunity, and inflammatory responses. This study examined the concentration of FFAR_2_ and found that it decreased along with that of acetic acid. Notably, FFAR_2_ expression may be associated with neurotoxicity and may be involved in brain development and neuronal differentiation as a potential drug target against several neurological diseases [37,38,39]. Schmidt et al. found that FFAR_2_ is mainly expressed on neutrophils, eosinophils, immune cells, and neuronal cells [40], but the neuronal cell function of FFAR_2_ has not been well explored. Razazan et al. indicated that the acetic acid-activated FFAR_2_-signaling pathway is associated with AD progression [10]. AD is the most common neurodegenerative disease in the elderly population [41],and has an increasing prevalence and no currently available effective preventive therapy or treatment. Amyloid β (Aβ) accumulation and formation of intracellular neurofibrillary tangles are characteristics of AD, causing neuronal changes and decreased memory function and learning capacity [42,43]. There is substantial evidence that neurodegeneration in AD pathology is caused by increased Aβ accumulation [44,45,46,47,48,49]. However, treatments targeting the reduction of Aβ levels have not yet been successful. A study by Kim et al. exploring the transition from wild-type mice to an AD mouse model of healthy microbiota transfer found that the decrease in the formation of Aβ plaques and neurogenic fibrous tangles was due to a change in the intestinal microbiota toward a normal trend, reduced neurotoxicity, and improved cognition, confirming the link between the gut and the brain in AD patients [50]. The results suggest that repression of the FFAR_2_-signaling pathway contributes to Aβ accumulation, increases Aβ-stimulated neuronal toxicity, and decreases neuronal cell survival. FFAR_2_ has also been confirmed to be richly expressed in human neuronal cells [10], and has been reported in human and mouse brain genome databases [51,52]. FFAR_2_ is also expressed in neuronal cells, indicating that it may have important effects on neuronal proliferation, differentiation, and activation, or other functions, further confirming that FFAR_2_ plays an integral role in neurons. These results show that the activation of FFAR_2_ may play a critical role in neuronal cells to reduce senescence and increase neuroprotection. Our study results indicated that the FFAR_2_ mRNA expression level was significantly reduced in the Cd group compared with that in the control group, suggesting that FFAR_2_ may be implicated in the regulatory mechanism of the gut–brain axis in Cd-induced neurodevelopmental toxicity.

## 5. Conclusions

Our study found that exposure at an environmentally relevant concentration of Cd (5 µg/L) altered the microbial composition and the concentrations of SCFAs. There was a positive correlation between acetic acid and *Phascolarctobacterium* and *Candidatus Saccharimonas* (*R* = 0.842, *p* < 0.01; *R*= 0.767, *p* < 0.01) and a negative correlation between isobutyric acid and *Blautia glucerasea* (*R* = −0.673, *p* < 0.05); all these bacteria were associated with neurotoxicity. Moreover, in terms of the mechanisms involved in the regulation of neuroprotective effects of FFAR_2_, FFAR_2_ must be activated first. SCFAs are one of its activators, and acetic acid in SCFAs is the major ligand of FFAR_2_. Our results indicated that compared with the control group, the concentration of acetic acid was decreased in the Cd group, which ultimately led to a decrease in FFAR_2_ expression in the Cd group. In summary, environmentally relevant concentrations of Cd can reduce the relative abundance of SCFA-producing intestinal flora, leading to a decrease in acetic acid concentrations, which in turn leads to a decrease in FFAR_2_ concentrations. Therefore, we hypothesize that Cd is implicated in the mechanism of neurodevelopmental toxicity through the gut–brain axis, demonstrating that FFAR_2_ might be a new potential target for the treatment of neurodevelopmental toxicity and neurodegenerative diseases.

## Figures and Tables

**Table 1:** List of primers used for gene expression.

Gene		Primer (5′-3′)	Accnumber	GeneID
*ffar*_2_	Forward	cgtcgcatttccaatccgat	NM_001082895.1	794045
	Reverse	tcacatggggattgagctgt		
*Gadph*	Forward	tctgacagtccgtcttgagaaa	NM_001115114.1	317741
	Reverse	acaaagtgatcgttgagagaa		

**Table 2:** Comparison of the short-chain fatty acid concentrations in zebrafish in the control group and Cd group.

	Average Concentration of Control Group (nmol/g)	Mean Concentration of Cd Group (nmol/g)	*p*
Acetic acid	51.8441	39.9531	0.09707
Isobutyric acid	0.04946	0.1141	0.03857
Isovaleric acid	0.03867	0.05075	0.3159
Valeric acid	0.09402	0.06351	0.4989
Hexanoic acid	0.5436	0.4975	0.7149

## Data Availability

The authors confirm that the data supporting the findings of this study are available within the article.
